# Safe threshold of capillary blood glucose for predicting early future neonatal hypoglycaemia in babies born to mothers with gestational diabetes mellitus, an observational, retrospective cohort study

**DOI:** 10.1186/s12884-021-03973-5

**Published:** 2021-07-09

**Authors:** Esther H. G. Park, Frances O’Brien, Fiona Seabrook, Jane Elizabeth Hirst

**Affiliations:** 1 Acute Medicine Department, Oxford University Hospitals NHS Foundation Trust, Oxford, UK; 2 Neonatology, Oxford University Hospitals NHS Foundation Trust, Oxford, UK; 3 Paediatrics, Oxford University Hospitals NHS Foundation Trust, Oxford, UK; 4 Nuffield Department of Women’s & Reproductive Health, Oxford University Hospitals NHS Foundation Trust, University of Oxford, Oxford, UK

**Keywords:** Gestational diabetes, Safe discharge, Neonatal hypoglycaemia

## Abstract

**Background:**

There is increasing pressure to get women and babies home rapidly after birth. Babies born to mothers with gestational diabetes mellitus (GDM) currently get 24-h inpatient monitoring. We investigated whether a low-risk group of babies born to mothers with GDM could be defined for shorter inpatient hypoglycaemia monitoring.

**Methods:**

Observational, retrospective cohort study conducted in a tertiary maternity hospital in 2018. Singleton, term babies born to women with GDM and no other risk factors for hypoglycaemia, were included. Capillary blood glucose (BG) testing and clinical observations for signs of hypoglycaemia during the first 24-h after birth. BG was checked in all babies before the second feed. Subsequent testing occurred if the first result was < 2.0 mmol/L, or clinical suspicion developed for hypoglycaemia. Neonatal hypoglycaemia, defined as either capillary or venous glucose ≤ 2.0 mmol/L and/or clinical signs of neonatal hypoglycaemia requiring oral or intravenous dextrose (lethargy, abnormal feeding behaviour or seizures).

**Results:**

Fifteen of 106 babies developed hypoglycaemia within the first 24-h. Maternal and neonatal characteristics were not predictive. All babies with hypoglycaemia had an initial capillary BG ≤ 2.6 mmol/L (Area under the ROC curve (AUC) 0.96, 95% Confidence Interval (CI) 0.91–1.0). This result was validated on a further 65 babies, of whom 10 developed hypoglycaemia, in the first 24-h of life.

**Conclusion:**

Using the 2.6 mmol/L threshold, extended monitoring as an inpatient could have been avoided for 60% of babies in this study. Whilst prospective validation is needed, this approach could help tailor postnatal care plans for babies born to mothers with GDM.

## Background

It is estimated that 17% of live births around the world are affected by hyperglycaemia in pregnancy, 84% of which have gestational diabetes mellitus (GDM) [1]. GDM is one of the commonest medical problems of pregnancy with increasing incidence worldwide [2]. Untreated, GDM is associated with maternal and neonatal complications [3]. One such complication, neonatal hypoglycaemia, mostly occurs within the first 24-h after birth as babies complete their metabolic transition over the first few days of life [4–6]. One observational study found that heel-prick plasma glucose concentrations of 2.6 mmol/L approximated the 10th percentile in the first 48-h but 39% of infants had ≥ 1 episode below this threshold [4]. Rarely, hypoglycaemia can bring about serious and long-lasting neurological sequelae if prolonged or recurrent [7] and there is particular concern for babies with mothers who had co-morbidities such as GDM.

Unfortunately, hypoglycaemia can be asymptomatic or accompanied by nonspecific symptoms. Therefore, screening programmes have been developed for early detection and management of hypoglycaemia and widely adopted amongst neonatologists. However, one of the limitations of such programmes is that asymptomatic euglycemic babies included in high-risk categories can experience excessive blood sampling, prolonged hospital admissions, and family separation [8, 9].

In 2017, the British Association of Perinatal Medicine (BAPM) published a framework for practice for the identification and management of neonatal hypoglycaemia in the full-term babies [10]. The BAPM framework was released in response to concerns about variable practice across the UK in the detection and management of hypoglycaemia. These pragmatic guidelines recommended a care pathway that includes early feeding and blood glucose (BG) monitoring, and regular assessment of clinical condition and feeding for 24 h for babies born to mothers with diabetes.

The adoption of the World Health Organisation (WHO) / International Association of Diabetes and Pregnancy Study Groups (IADPSG) criteria to diagnose GDM has resulted in more women being diagnosed with GDM, with consequently more babies monitored on a high-risk pathway for hypoglycaemia. Monitoring increases workload for midwives, requires in-patient stay for the woman for at least 24-h, and requires heel-prick testing timed before feeds for the baby.

In response to queries from mothers in our hospital about the need for inpatient monitoring, we sought to determine whether in our local population, the risk of neonatal hypoglycaemia could be predicted in babies born to women with GDM, and whether a group of babies could be identified as low-risk and potentially safe to go home earlier.

## Methods

This was an observational retrospective cohort study using routinely collected clinical data conducted in a teaching hospital in South East England, delivering approximately 7,500 babies per year. Any incidences of hospital re-admission were reviewed up to 6 weeks post-partum. Data analysis occurred between February 2019 and March 2020 and validation data analysis between August and September 2020.

We included singleton, liveborn, term babies (≥ 37 completed weeks) born to mothers with GDM born between August-December 2018 with at least one BG reading documented in the first 24-h of life. GDM was diagnosed following National Institute for Health and Care Excellence (NICE) [11] 2015 clinical risk factor screening criteria, with IADPSG diagnostic criteria for the 75 g oral glucose tolerance test (OGTT). We excluded babies with major congenital abnormalities, those requiring immediate neonatal intensive care unit (NICU) admission, and babies in whom hypoglycaemia monitoring would be recommended for another reason (e.g. fetal growth restriction, birthweight for gestational age < 2^nd^ centile, beta-blocker therapy during pregnancy, neonatal sepsis).

We assessed the risk of neonatal hypoglycaemia in relation to: maternal characteristics (age, Body Mass Index (BMI), parity, non-white ethnic group), treatments for diabetes during pregnancy (diet, metformin, insulin), use of insulin infusion during labour, birthweight for gestational age and gender according to The International Fetal and Newborn Growth Consortium for the 21^st^ Century [12] criteria, time to first feed, mode of feeding (artificial or breast), and first capillary BG.

### Primary outcome

In order to capture all babies potentially with clinically significant hypoglycaemia, we adopted a composite definition of either BG ≤ 2.0 mmol/L, measured by heel-prick test or a ward-based blood gas machine using a whole blood capillary sample and/or clinical symptoms (lethargy, abnormal feeding behaviour, or seizures) requiring buccal or IV dextrose within the first 24-h of life.

Our trust guideline at the time of this study recommended all babies have at least one BG measurement before the second feed. If the first BG was ≥ 2.6 mmol/L and baby was feeding well, he was observed for clinical signs of hypoglycaemia for 24-h as an in-patient. If the first BG was ≤ 2.6 mmol/L, or he showed possible signs associated with hypoglycaemia, or was feeding poorly, further pre-feed tests were performed.

Neonatal BG were measured using capillary point-of-care testing with Freestyle Precision Pro (Abbott Diabetes Ltd. FCC ID: N6C-SXSDCAG). When the heel-prick BG was low, capillary blood was tested on a blood gas analyser (Radiometer ABL) for confirmation.

The main source of data was maternal and neonatal records including electronic patient records (Cerner Millennium and Badger) and handwritten clinical notes.

Women with GDM who delivered between 6/8/18 and 31/12/18 were included in the study. Verification was performed on babies born between 1/1/19 and 4/6/19 who met the same entry criteria. As this was an exploratory study, no formal sample size calculation was performed.

### Statistical methods

Pregnancy, delivery and neonatal characteristics for each group were described using mean and standard deviation for normally distributed data, median and range for non-normally distributed data, and number and percentage for categorical data. Continuous variables were assessed for normality and if required, log transformed.

Univariate unconditional logistic regression was used to assess the association between clinical characteristics and neonatal hypoglycaemia. Statistical significance was considered at 0.05. Fisher’s Exact Test was used to compare categorical outcomes. The optimal threshold of the first BG to identify babies at risk of neonatal hypoglycaemia in the first 24-h of life was determined using the Area under the ROC Curve (AUC), with the threshold chosen at 100% sensitivity. We validated this through the AUC derived from the first BG of babies born in the next six months in our hospital to women meeting the inclusion criteria, ten of whom developed hypoglycaemia.

Missing data were excluded from analysis.

Statistical Package for the Social Sciences (SPSS) Statistics software v25 was used for all statistical procedures.

## Results

**Fig. 1:**
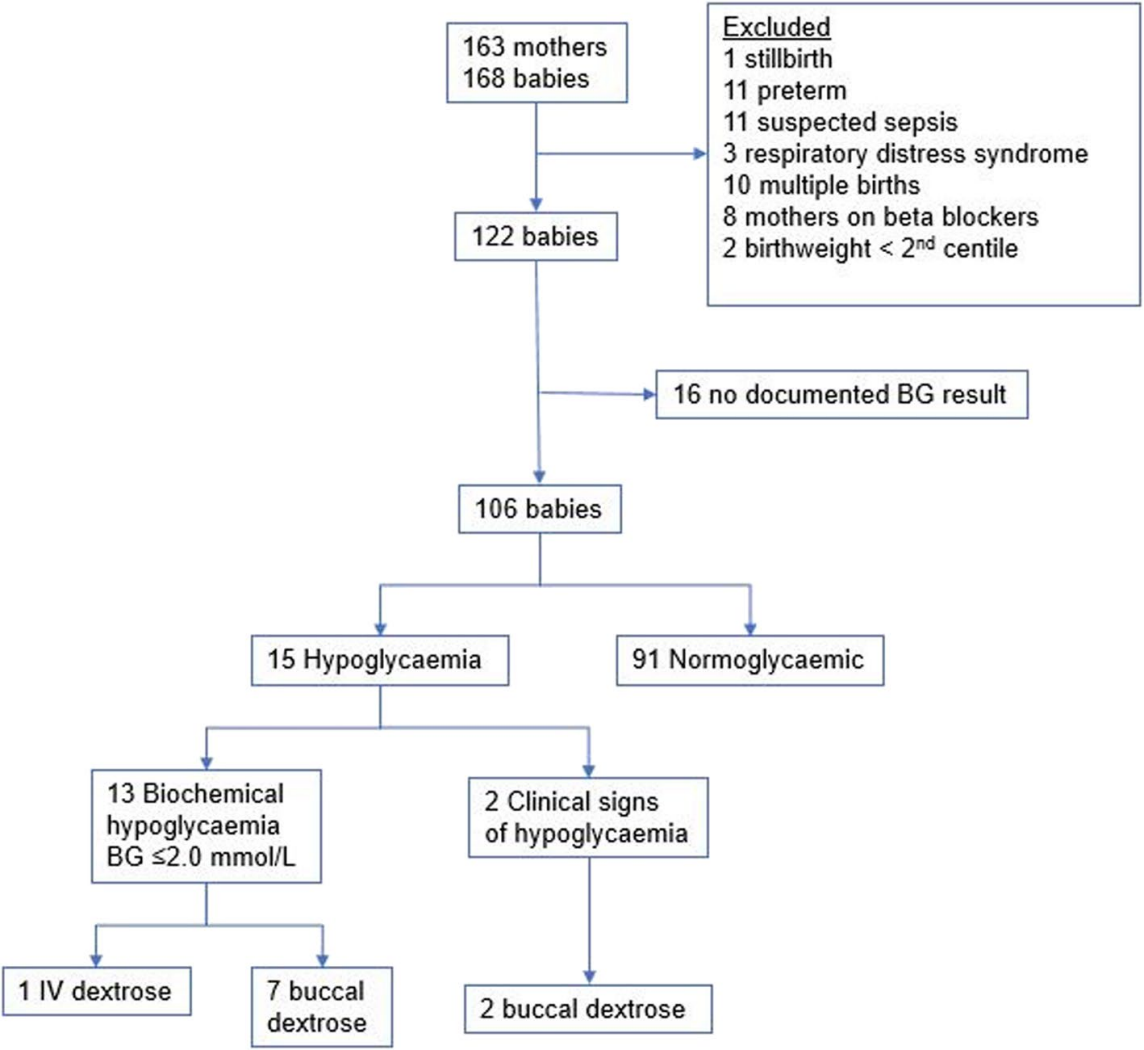
Flowchart of included babies and number of blood glucose treatments during their in-patient observation period

From 6/8/18 to 31/12/18, 168 babies were born to 163 mothers with GDM. Of these, we excluded 62 babies: 11 preterm, 11 admitted to NICU with suspected sepsis, 10 twins, 3 with respiratory distress, one stillbirth, 8 born to mothers taking beta-blockers, two with birth weight < 2nd centile and, and 16 without a documented BG. Thus, 106 babies were included in the analysis (Fig. 1).

Characteristics of babies and their mothers are reported in Table 1. Of note, half the mothers were obese (*n* = 56, 52%), with just under half needing pharmacologic treatment for their GDM (*n* = 49, 46%). The majority of deliveries were vaginal (*n* = 75, 69%). One woman developed pre-eclampsia; 46 had blood loss > 500 mL at delivery (23 vaginal deliveries, 23 Caesarean sections), and 7 women developed peripartum sepsis.

**Table 1:** Maternal baseline, GDM, labour and birth, and newborn characteristics

Characteristic	N	Total	Babies with hypoglycaemia^a^, ^b^	Babies with no hypoglycaemia	*P* value
**Mother**					
Age in years	106	Mean 32.3 (range 19–45)	32.6 years	32.3 years	0.8
BMI in kg/m^2^	106				
Normal (18.5–25)		19 (18%)	2	17	-
Overweight (25.01–30)		32 (30)	3	29	0.9
Obese (> 30)		55 (52)	10	45	0.4
Parity	106				
0		45 (41)	7	38	-
1 or more		61 (59)	8	53	0.8
Ethnicity	106				
White Caucasian		59 (54)	10	49	-
Non-white		47 (46)	5	42	0.5
**GDM**					
OGTT mmol/L					
fasting	99	5.1 (4.0–11.5)	5.5 mmol/L	5.1 mmol/L	0.3
1-h	93	9.7 (4.1–15.5)	10.3	9.6	0.2
2-h	95	7.4 (3.8–13.5)	7.6	7.4	0.8
HbA1c (%)	100	5.2 (4.0–6.6)	5.2%	5.2%	0.7
Diabetes control	106				
Diet alone		56 (52)	7	49	-
Metformin		35 (34)	6	29	0.5
Insulin (± metformin)		13 (12)	2	11	0.9
**Labour and delivery**					
Onset of labour	106				
Spontaneous		41 (38)	4	37	-
Induced		44 (42)	8	36	0.3
No labour		21 (20)	3	18	0.6
Intrapartum BG	99				
Self		91 (92)	11	80	-
Variable rate insulin intravenous infusion		8 (8)	2	6	0.3
Mode of delivery	106				
Vaginal spontaneous		60 (57)	7	53	
Vaginal assisted		14 (13)	3	11	0.3
Caesarean section		32 (30)	5	27	0.6
**Neonatal characteristics**					
Sex	106				
Male		58 (55)	8	50	0.9
Gestational age at delivery	106	275 days (range 259–290)	278	275	0.1
Birthweight	106				
> 90th centile		24 (23)	0	24	-
< 10th centile		5 (5)	0	5	-
**Feeding characteristics**					
Mode of first feed	103				
Breast		92 (89)	14	78	0.4

^a^Hypoglycaemia as per pragmatic definition including biochemical and clinical signs. ^b^ *N unless units given*

The mean gestational age at birth was 39.3 weeks and mean birth weight was 3455 g (Standard Deviation (SD) 486 g). The first feed was breastmilk for 93% of babies.

All 106 babies had a BG measurement before their second feed. After the first BG, 38 babies had one further measurement (two measurements in total), 20 babies had two further measurements, and 12 babies had more than three further BG measurements.

### Patterns of Neonatal hypoglycaemia

Thirteen babies developed measured hypoglycaemia (BG ≤ 2.0 mmol/L) in the first 24-h and a further two babies were treated with buccal dextrose due to clinical signs of hypoglycaemia (i.e. 15 babies in total with clinical or biochemical hypoglycaemia). Five recovered without any treatment. One of these babies had hypoglycaemia persisting after 24-h of age despite treatment with buccal dextrose. There were no cases of severe hypoglycaemia (BG ≤ 1.0).

Out of the 13 babies with measured hypoglycaemia in the first 24-h, 11 were detected at the first BG, one was detected at the second test, and one at the third test. Both these babies with later detection had initial BG ≤ 2.6 and according to the protocol in place at the time, they had the second and third BG done. It was noted that both mothers of the babies detected at the second and third tests had significant psychiatric co-morbidities.

There were two cases of persistent hypoglycaemias (BG ≤ 2.0 mmol/L detected on three or more measurements). One of these babies was identified at first BG (1.4 mmol/L) and admitted to neonatal unit for intravenous (IV) dextrose. The other baby had persistent hypoglycaemia after 24-h. Her mother had a history of anxiety and other comorbidities who had declined antenatal breast-feeding support. She was admitted to NICU for monitoring and recovered with buccal dextrose.

The remaining 91 babies had neither biochemically recorded hypoglycaemia nor received treatment for clinical suspicion of hypoglycaemia.

Nine babies were admitted to NICU, including four (of the 15) who had hypoglycaemia, and one for hypoxia, jaundice, bradycardia, poor tone and maternal sepsis respectively.

There were 11 readmissions during the first 6 weeks of life but no readmissions due to hypoglycaemia.

### Clinical risk factors for neonatal hypoglycaemia

No maternal, birth or neonatal risk factors were associated with neonatal hypoglycaemia in this study population (Table 1), therefore multivariate analysis was not performed.

**Fig. 2:**
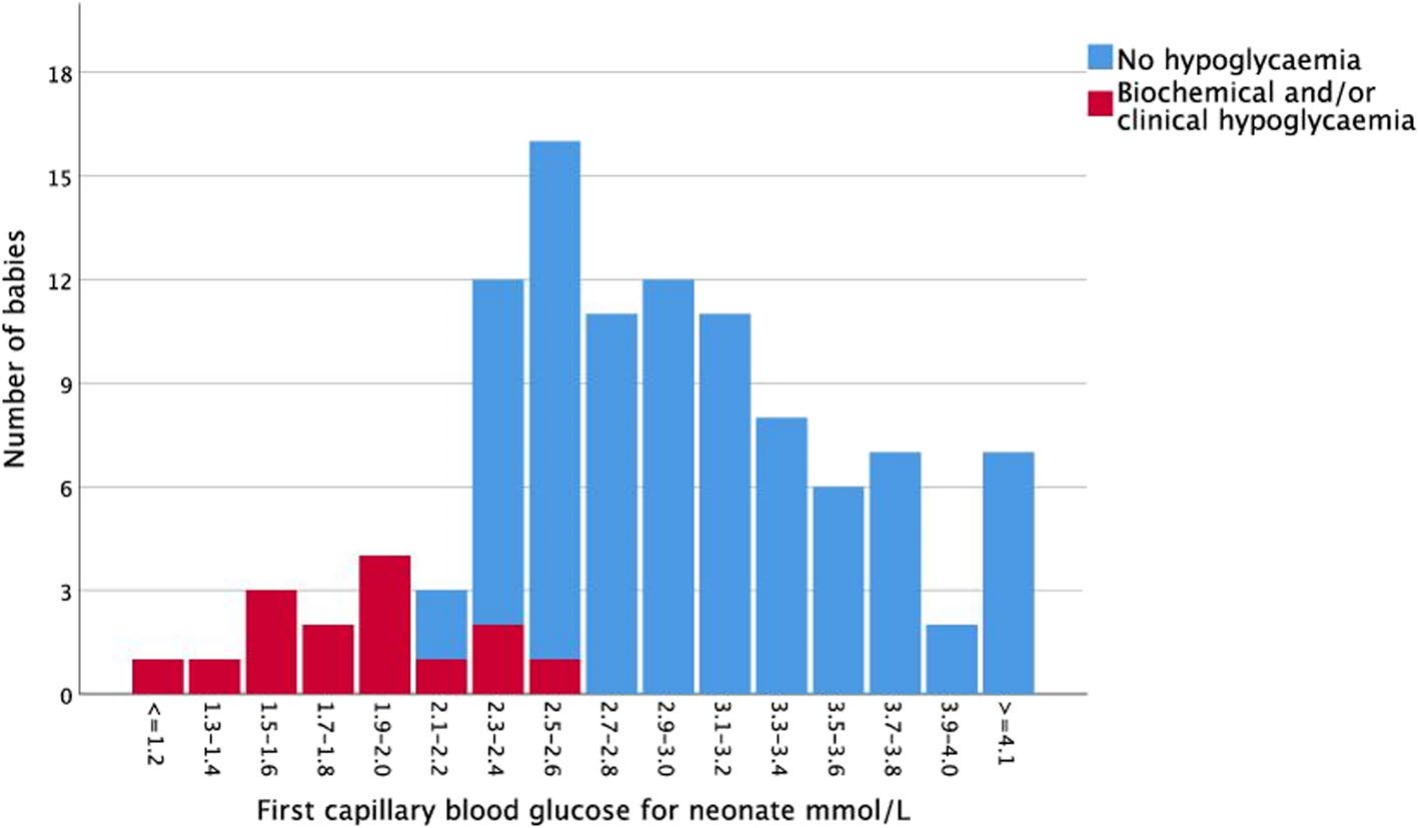
First neonatal capillary blood glucose reading and subsequent biochemical or clinical hypoglycaemia

**Fig. 3:**
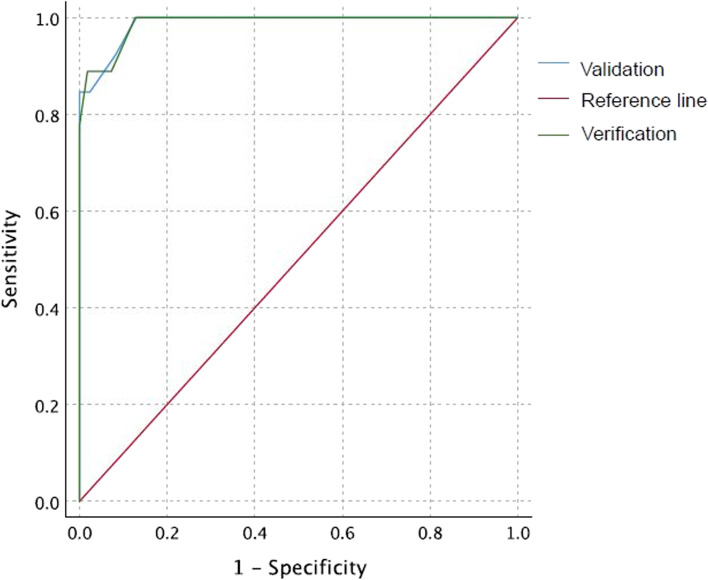
Area Under the Curve (AUC) for validation study

The results of the first capillary BG result for babies performed before the second feed are presented in Fig. 2. There were 42 babies with a reading ≤ 2.6 mmol/L at the first test. This included all 15 babies diagnosed with BG ≤ 2.0 mmol/L, either at this test (11 babies), or subsequently (4 babies) within the first 24-h, with biochemical or clinical hypoglycaemia. The AUC was 0.96 (95% CI 0.91–1.0) for the first neonatal BG to predict hypoglycaemia at any time during the first 24-h of life. A threshold of 2.6 mmol/L achieved 100% sensitivity, i.e. all babies with hypoglycaemia at any time in the first 24-h had an initial BG of 2.5 mmol/L or less. This result was validated in a further 65 babies born to mothers with GDM without other risk factors for hypoglycaemia who met the study entry criteria, AUC 0.99 (95% CI 0.96–1.0) (Fig. 3).

Each baby with one or more episodes of hypoglycaemia had on average 4.5 tests performed, compared to 1.7 tests in the whole cohort (range 1–6), suggesting that babies at risk were correctly identified and monitored more intensely.

## Discussion

We demonstrate in babies born to mothers with GDM, maternal, neonatal, birth or early feeding characteristics are unreliable predictors of neonatal hypoglycaemia. However, by using the threshold of 2.6 mmol/L at the first neonatal BG, all babies that subsequently developed neonatal (clinical or biochemical) hypoglycaemia in the first 24-h of life were identified. The BAPM recommends a threshold of intervention for hypoglycaemia at < 2.0 mmol/L. Whilst our data do not challenge this, as a screening test to identify newborns at low risk, 2.6 mmol/L appears to perform well in our babies. We used a pragmatic definition of neonatal hypoglycaemia as we aimed to capture clinically relevant events that would be best managed in hospital. We have adopted this approach both to allow for the well-recognised variability in the handheld capillary BG meters particularly at low readings and recognising the likely minimal long-term effects of very transient episodes of hypoglycaemia in otherwise well newborns. This study suggests that effective feeding can be challenging to establish, and additional support may be required for women with underlying psychiatric conditions.

The BAPM framework recommends interventions to raise BG in babies with a “BG < 1.0 mmol/L; a single value of < 2.5 mmol/L in a baby with abnormal clinical signs; or a value of < 2.0 mmol/L and remaining < 2.0 mmol/L at next measurement in a baby with risk factors for metabolic adaptation.” The BAPM screening guideline is the same for all babies at risk of hypoglycaemia regardless of the underlying metabolic condition. Our study demonstrates that there may be a group of babies born to mothers with GDM who are at lower risk of developing clinically significant hypoglycaemia. If the threshold of 2.6 mmol/L for the first BG had been used as a screening test in these babies, it had a sensitivity of 100% (95% CI 74-100%) to correctly identify the babies at risk of subsequent hypoglycaemia, and specificity of 69% (59–79%). This suggests that if babies have a first BG > 2.6 mmol/L, they would not require a second BG prior to third feed as recommended by BAPM unless they developed signs of hypoglycaemia.

The Coronavirus disease (COVID-19) pandemic has resulted in many maternity units in the UK adopting a model of care aiming for earlier discharge of women and babies to minimise exposure to the virus. All mothers should be given education on the signs of poor feeding and possible hypoglycaemia in their newborn. The decision on whether mothers can go home to continue this monitoring or need a full 24-h inpatient monitoring needs to be carefully considered. For mothers with physical or mental conditions impairing their ability to feed may need close inpatient observation. This study could help guide which babies born to mothers with GDM could be safely managed in the community, reducing the time spent in hospitals for women and babies who would otherwise be able to go home.

The IADPSG criteria for GDM were based on the Hyperglycemia and Adverse Pregnancy Outcome (HAPO) study [13], which demonstrated linear increases between worsening hyperglycaemia at the 28-week OGTT, with the risks of birth weight > 90^th^ centile, primary Caesarean-section, and cord blood serum Connecting-peptide (C-peptide). A linear relationship with clinical neonatal hypoglycaemia was weak, and the impact of changing to these criteria on neonatal inpatient monitoring is significant. A number of studies have reported higher rates of hypoglycaemia and intravenous dextrose use in babies born to insulin-treated women compared to women treated by metformin or diet [14–16]. Conversely, other groups have demonstrated that there is no real difference between maternal treatment modalities [5, 6]. Our study supports these findings, namely that the risk of neonatal hypoglycaemia is not predictable from maternal background characteristics, medication requirement for GDM, or the birthweight (excluding babies born < 2^nd^ centile).

### Strengths and limitations

This was a pragmatic study conducted in a busy hospital setting. We captured everyday practice. Our population has similarities to other maternal populations in the UK and other high-income settings, with high rates of obesity observed.

Our study’s most notable limitation was our small sample size. As a result, we had limited power to detect differences in rarer outcomes, such as severe neonatal hypoglycaemia. As we did not have data on repeat BG for all neonates for the full 24-h, we cannot exclude that some babies experienced biochemical hypoglycaemia which was not severe enough to manifest in symptoms. The significance of this in babies that are otherwise alert and feeding well has been questioned [10]. All babies were however observed in hospital for at least 24 h. The threshold of 2.6 mmol/L was consistent with our previous clinical guideline. We present data supporting that in babies born to women with GDM without other risk factors for hypoglycaemia, if the first BG is > 2.6 further measurements are not required. This could be considered in the future as a threshold for a low-risk pathway.

We relied on routine clinical documentation to ascertain the primary outcome. Some mothers and babies had no BG recordings documented, which could have been due to poor documentation or omission of the test. This reduced our sample size further. However, none of those babies were admitted to NICU for suspicion or management of hypoglycaemia.

Handheld point-of-care glucometer used in the clinical setting has known inaccuracies compared to laboratory methods, with false hypoglycaemia being more common than false normoglycaemia [9]. In the case of newborns, the haematocrit also plays a role. However, we wanted the means of BG testing to reflect every day practice in the majority of the maternity units across the country. We surmised that given the convenience of glucose point-of-care testing, many units across the nation rely on BG result from handheld glucometers as the first-line clinical tool for detecting neonatal hypoglycaemia without adjustment to haematocrit. Furthermore, as we tested for ‘safe upper threshold’ rather than ‘treatment threshold’, we believe that the inaccuracies at low readings affect our conclusion little.

## Conclusion

Acknowledging the limitation of the small sample size, and known inaccuracies of handheld glucometer, we demonstrate that whilst maternal characteristics of women did not predict which babies developed neonatal hypoglycaemia, if the first neonatal BG is > 2.6 mmol/L, the baby was subsequently at low-risk of biochemical or clinical hypoglycaemia. If a strategy of no further testing were adopted for babies in our unit with this threshold, this could reduce the need for further capillary tests and monitoring for around two-thirds of babies. Whether this group of mothers and babies are truly ‘safe to go home’ will depend on the feeding pattern and capacity of the mother to monitor her baby. This approach needs to be used with vigilance in particular high-risk groups (maternal physical and mental health co-morbidities).

## Data Availability

Original data will be available from corresponding author upon reasonable request.

## Abbreviations

GDM: Gestational Diabetes Mellitus
BG: Blood Glucose
AUC: Area Under the ROC Curve
CI: Confidence Interval
BAPM: British Association of Perinatal Medicine
WHO: World Health Organisation
IADPSG: International Association of Diabetes and Pregnancy Study Groups
NICE: National Institute for Health and Care Excellence
OGTT: Oral Glucose Tolerance Test
NICU: Neonatal Intensive Care Unit
BMI: Body Mass Index
INTERGROWTH-21^st^: The International Fetal and Newborn Growth Consortium for the 21^st^ Century
SPSS: Statistical Package for the Social Sciences
SD: Standard Deviation
IV: Intravenous
COVID-19: Coronavirus Disease
HAPO: Hyperglycaemia and Adverse Pregnancy Outcome
C-peptide: Connecting-peptide
